# The Functional Outcome of Limb Salvage of Proximal Femur Tumors With Modular Endoprosthesis: A Prospective Study

**DOI:** 10.7759/cureus.50375

**Published:** 2023-12-12

**Authors:** Hiranya Kumar, Karthik N Mittemari, VamshiKrishna Chand Nimmagadda, Ajith T Abraham, Shivaraj Nadagouda, Deepank Choudry

**Affiliations:** 1 Trauma and Orthopedics, Vydehi Institute of Medical Sciences and Research Centre, Bangalore, IND; 2 Orthopedics, Vydehi Institute of Medical Sciences and Research Centre, Bangalore, IND; 3 Orthopedic Surgery, Vydehi Institute of Medical Sciences and Research Centre, Bangalore, IND

**Keywords:** bone tumor and limb salvage, functional outcome, msts, proximal femur tumors, modular endoprosthesis

## Abstract

Introduction

The proximal femur is a common site for primary bone sarcomas, including Ewing's sarcoma, chondrosarcoma, osteosarcoma, and giant cell tumors (GCT). Extensive resections are challenging to reconstruct because the size of the tumor may necessitate an extensive resection of the femur to achieve adequate oncologic clearance. The resection of the proximal femur can result in hip joint instability due to the loss of the strong native hip capsule or hip abductor strength. With a wide range of reconstruction options, such as resection arthrodesis, allograft-prosthetic composite (APC), and endoprosthesis, we can achieve limb salvage with good functional outcomes.

Objective

The objective of this case series is to evaluate the clinical outcomes and complications associated with endoprosthesis replacement in patients with proximal femur tumors.

Methods

A prospective analysis was conducted with 32 patients who underwent modular endoprosthesis replacement for proximal femoral tumors at our institution. Patient data, tumor characteristics, surgical details, and postoperative outcomes were collected and analyzed.

Results

The study involved 32 patients who met the inclusion criteria. They were assessed using the Musculoskeletal Tumor Society Score (MSTS), which showed good functional outcomes in 72.84% of the patients. The study highlights the functional outcomes and potential complications associated with the use of modular prostheses.

Conclusion

Endoprosthesis replacement in proximal femur tumors is a viable treatment option, providing good functional outcomes and an acceptable method for limb salvage, which enhances the quality of life.

## Introduction

The proximal femur is a common site of primary bone sarcomas, with 16% of Ewing's sarcomas, 13% of chondrosarcomas, 10% of osteosarcomas, and 5.5% of giant cell tumors (GCT), as stated by Bickels et al. [1]. Previously, amputation remained the mainstay of treatment. With improved comprehension and expanding medical knowledge, treatment guidelines have contributed to an increase in limb salvage procedures. Although extensive resections pose a challenge for reconstruction, the size of the tumor may necessitate extensive resection of the femur to achieve adequate oncologic clearance. The resection of the proximal femur can result in hip joint instability due to the loss of the strong native hip capsule or hip abductor strength. With a wide range of reconstruction options, such as resection arthrodesis, allograft-prosthetic composite (APC), reimplantation of tumor-bearing bone, and endoprosthesis, we can achieve limb salvage with good functional outcomes.

The approach to limb salvage includes a thorough evaluation of the tumor including its type and staging, age, gender, general condition, overall functionality, and quality of life. Modular endoprosthesis is an acceptable method for managing proximal femur tumors [2,3]. It is a limb salvage technique that enhances the quality of life. In addition, it is widely used because it offers intraoperative flexibility, easy availability, and early mobilization; does not interfere with adjuvant therapy; and is cost-effective. Complications of the procedure have been widely documented, such as infection, dislocation, aseptic loosening, implant breakage, skin necrosis, periprosthetic fractures, and other complications, including neurovascular injuries, pulmonary embolism, and deep vein thrombosis. Functional outcomes of proximal femur megaprosthesis fixation have been measured using the Musculoskeletal Tumor Society Score (MSTS), which is widely accepted and used in the present study.

## Materials and methods

Aim

The study aims to evaluate the functional outcome and complications associated with resection and modular endoprosthetic replacement in proximal femur tumors.

Source of the data

A prospective study involving 32 patients was conducted at our institution, a tertiary-level healthcare center, from 2012 to 2019, with a mean follow-up period of five years. There were 14 males and 18 females, and the mean age was 31 years. We have encountered various types of proximal femur tumors, including 17 cases of giant cell tumors (53.1%), nine cases of osteosarcomas (28.1%), five cases of secondary tumors (15.6%), and one case of fibrous dysplasia (31.2%), for which limb salvage was done using endoprosthesis.

Method of data collection

On admission of the patient, a careful history was elicited from the patient and/or their attendants regarding the course of the disease. The general condition of the patient and the vital signs were recorded. The examination was conducted systematically to rule out metastasis. The affected side was examined locally and compared with the normal contralateral side. Deformity, loss of function, altered attitude, any nerve injury, and vascular compromise were also looked for and noted. The patient was taken for surgery after a thorough preoperative evaluation, which included modalities such as X-rays, MRIs, PET scans, and blood investigations. Consent for surgery was also obtained from the patient and their attendants after explaining the procedure and possible complications. Tumors are classified [4,5] according to the Enneking system.

Postoperative adjuvant therapy was followed according to the protocol to evaluate the functional outcome. Patients were followed up on a monthly basis during the first six months, every three months during the next six months, and every six months thereafter.

Patients with proximal femur tumors were included in the study, while patients with tumors involving the bones, nerves, vessels, and soft tissue in all three components were excluded.

Operative procedure

The surgical procedure consists of three stages: tumor excision, endoprosthesis placement, and soft tissue coverage. A thorough preoperative plan was done, followed by a predetermined bone and soft tissue resection as planned by CT or MRI scans. The tumor resection involved the extensive removal of the tumor margins, ensuring wide free margins for endoprosthesis fixation. A standardized posterolateral approach was used for the resection of proximal femur tumors. After a wide excision of the tumor, the endoprosthesis was positioned after reaming and fixed, which offered appropriate rotational alignment based on anatomical landmarks. Movements were assessed intraoperatively. Adequate soft tissue coverage was achieved.

Postoperatively, an abduction brace was secured with adequate antibiotic coverage until the drain tubes were removed. Mobilization was done with the abduction brace for the initial two weeks, and range of motion (ROM) exercises were initiated from day 3 of surgery within tolerable limits. The brace continued until active abduction was achieved before full weight-bearing mobilization without the brace.

Treatment

Patients with osteosarcoma received neoadjuvant chemotherapy and two cycles of methotrexate, doxorubicin, and cisplatin, followed by surgery. Postoperatively, either four cycles of methotrexate, doxorubicin, and cisplatin or three cycles of ifosfamide, doxorubicin, and cisplatin were administered. Giant cell tumor and chondrosarcoma patients were treated only with surgery.

## Results

The functional outcome in 32 patients showed a good and satisfactory outcome, which was 72.84%, according to the MSTS. According to the MSTS, we analyzed the functional outcomes in terms of gait, walking, support walking, emotional, function, and pain after a five-year follow-up period.

**Figure 1:**
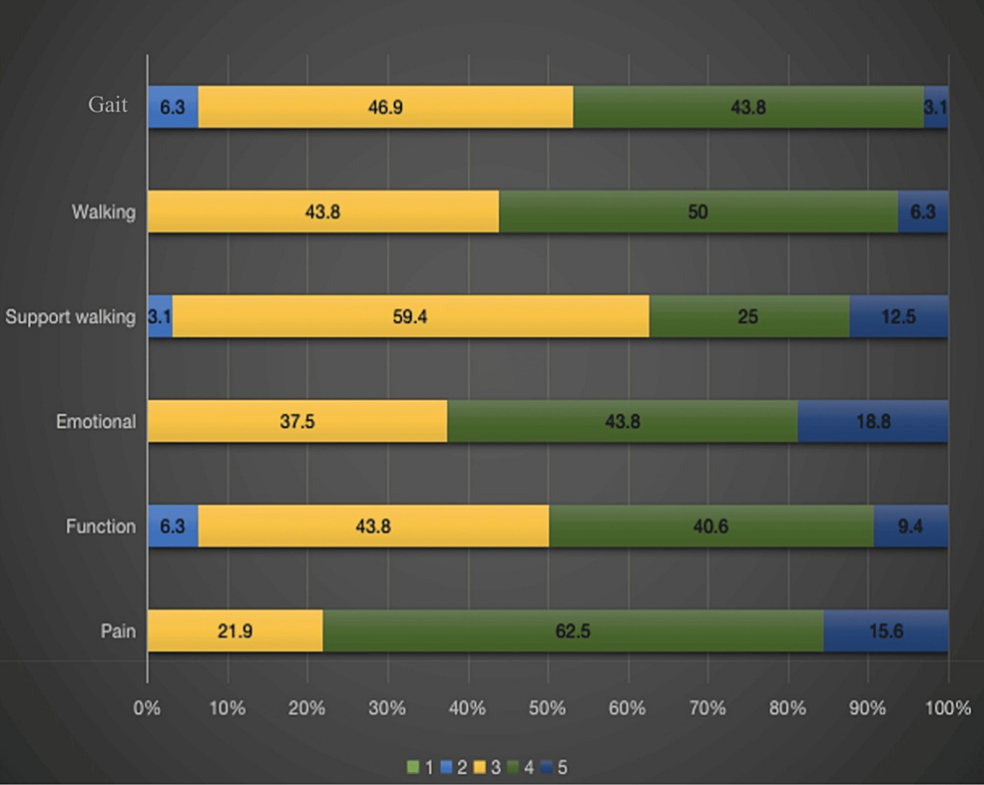
Graphical Representation of the Results of Musculoskeletal Tumor Society Score Assessment.

The graphical data of the MSTS are represented in Figure 1: under the parameters of gait: two patients (6.3%) with a score of 2, 15 patients (46.9%) with a score of 3, 14 patients (43.8%) with a score of 4, and one patient (3.1%) with a score of 5; under the parameters of walking: 14 patients (43.8%) with a score of 3, 16 patients (50%) with a score of 4, and two patients (6.3%) with a score of 5; under the parameters of support walking: one patient (3.1%) with a score of 2, 19 patients (59.4%) with a score of 3, eight patients (25%) with a score of 4, and four patients (12.5%) with a score of 5; under the parameters of emotional: 12 patients (37.5%) with a score of 3, 14 patients (43.8%) with a score of 4, and six patients (18.8%) with a score of 5; under the parameters of function: two patients (6.3%) with a score of 2, 14 patients (43.8%) with a score of 3, 13 patients (40.6%) with a score of 4, and three patients (9.4%) with a score of 5; and under the parameters of pain: seven patients (21.9%) with a score of 3, 20 patients (62.5%) with a score of 4, and five patients (15.6%) with a score of 5. The scoring was done according to the MSTS, with an overall score of 72.84%, corresponding to a good functional outcome.

Case 1

**Figure 2:**
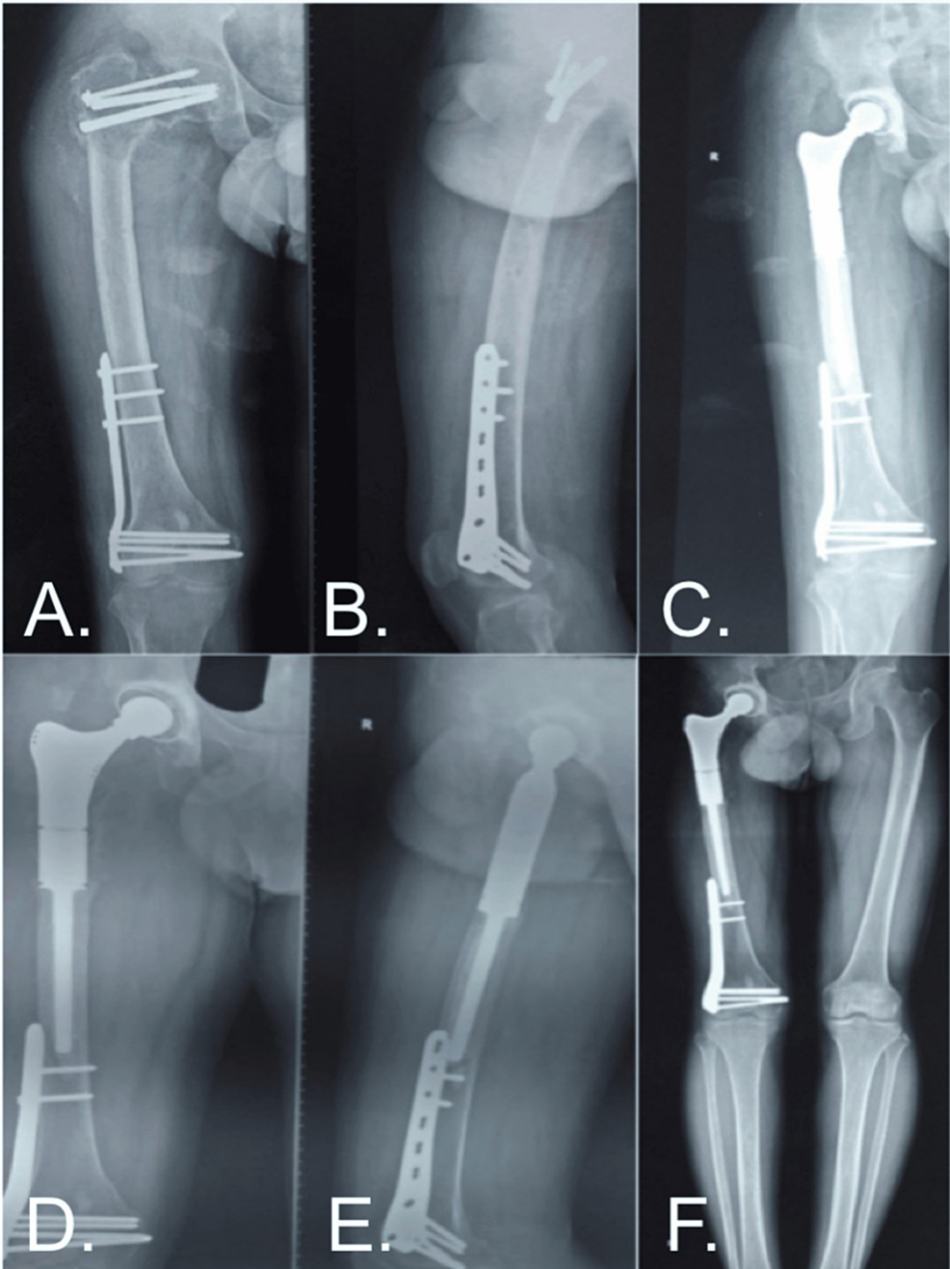
A Case of a 38-Year-Old Male With Recurrent GCT of the Right Proximal Femur. GCT: giant cell tumor

A 38-year-old male patient presented with a radiograph (Figure 2A, 2B) of the right femur showing an osteolytic lesion in the proximal region. There were cannulated cancellous (cc) screws in situ, proximally fixed for the right neck of the femur fracture, and a right lateral distal femur plate in situ. After a thorough evaluation and pre-anesthetic clearance, the patient underwent limb salvage surgery with the right proximal femur tumor excision and endoprosthesis fixation (Figure 2C). Radiographs (Figure 2D, 2E) and a scannogram (Figure 2F) were taken after 36 months of follow-up.

Case 2

**Figure 3:**
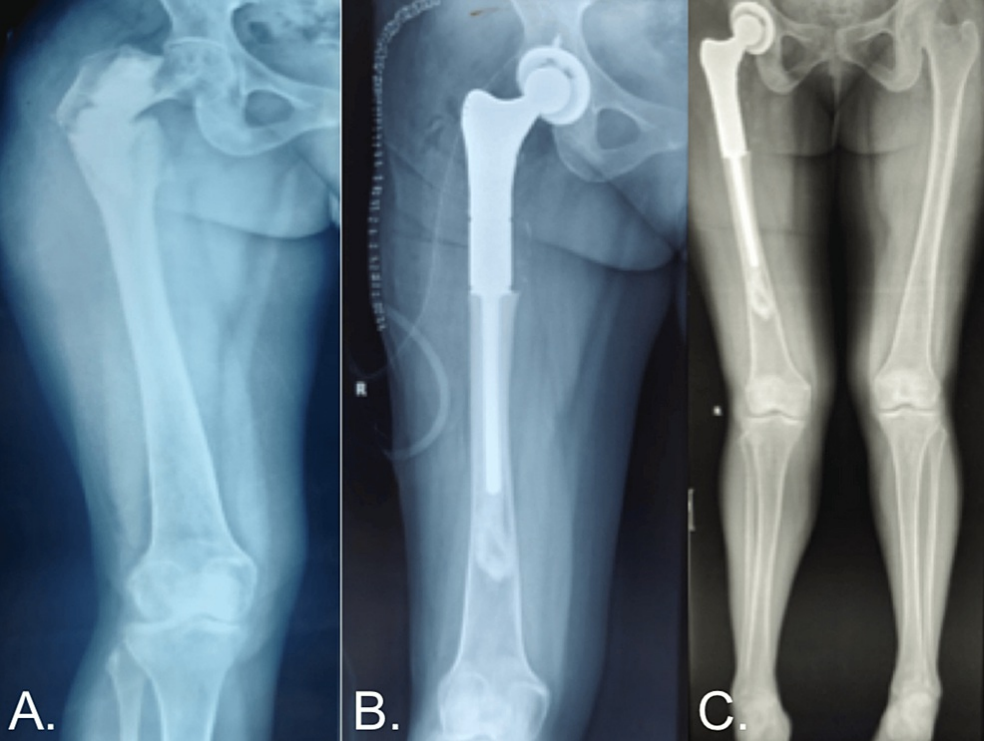
A Case of a 43-Year-Old Female With Fibrous Dysplasia of the Right Proximal Femur.

A 43-year-old female diagnosed with fibrous dysplasia of the right proximal femur presented with a pathological fracture of the right proximal femur (Figure 3A). Pre-anesthetic fitness was assessed, and the patient underwent limb salvage surgery on the right proximal femur. A radiograph (Figure 3B) was taken after tumor resection and endoprosthesis application. A lower limb scannogram (Figure 3C) was performed during a 12-month follow-up.

Case 3

**Figure 4:**
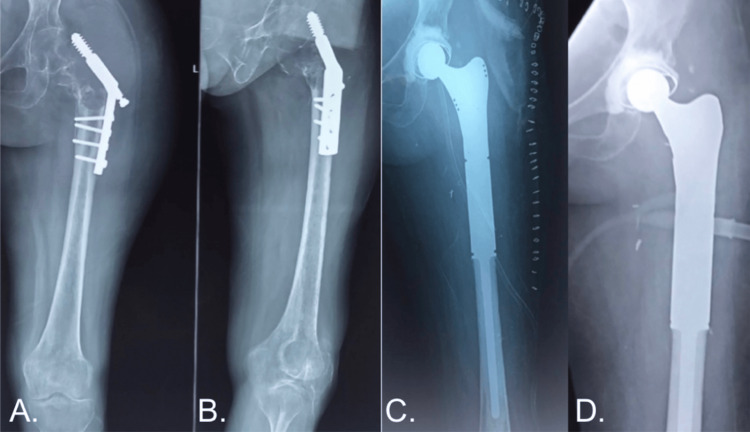
A Case of a 41-Year-Old Female Diagnosed With Metastasis Secondary to Carcinoma of the Thyroid.

A 41-year-old female patient with a known history of thyroid carcinoma and prior dynamic hip screw fixation presented with complaints of pain in the left hip. On further radiologic evaluation, an osteolytic lesion (Figure 4A, 4B) was noted on the left proximal femur with an implant in situ. The patient underwent limb salvage after receiving pre-anesthetic clearance. The postoperative radiograph (Figure 4C) shows the proper positioning of the endoprosthesis. The patient showed an improvement in the MSTS during regular follow-up, and a radiograph (Figure 4D) was done after a two-month follow-up.

Case 4

**Figure 5:**
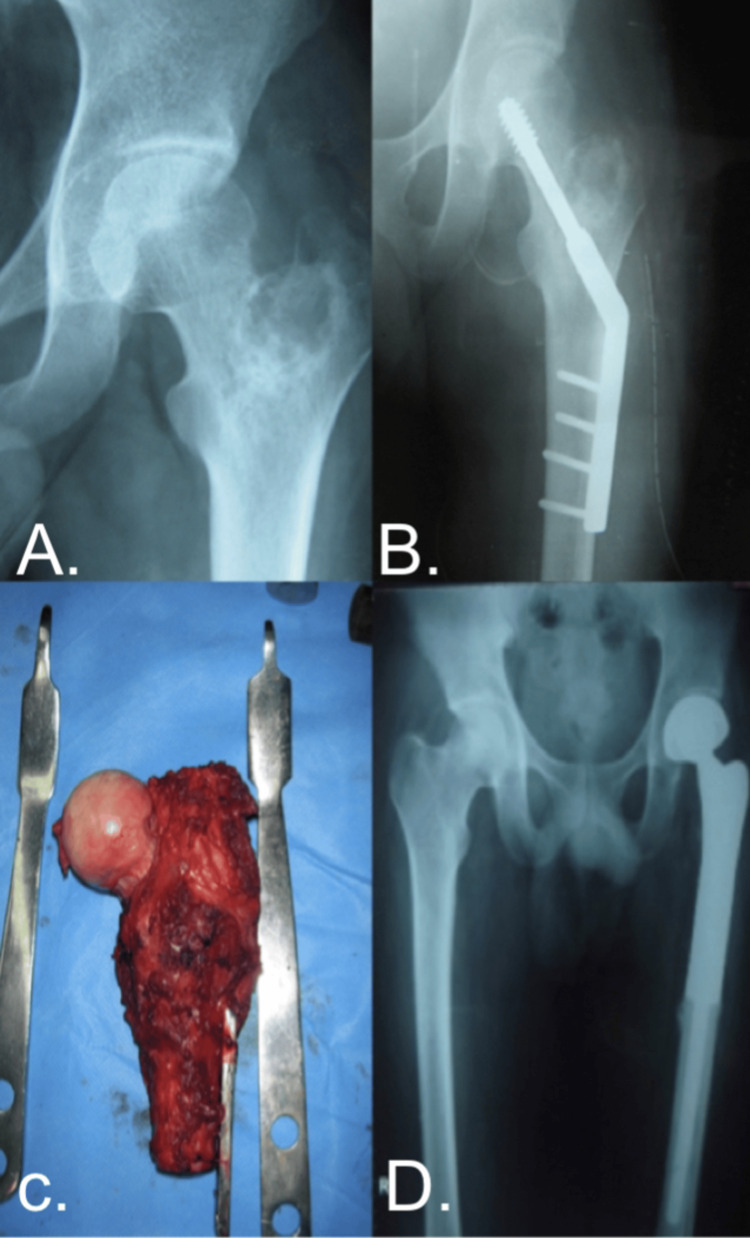
A Case of a 23-Year-Old Male With Telangiectatic Osteosarcoma.

A 23-year-old male presented with left hip pain. An osteolytic lesion (Figure 5A) was noted on the initial radiograph of the left proximal femur. The patient underwent curettage and prophylactic dynamic hip screw fixation (Figure 5B). A biopsy revealed telangiectatic osteosarcoma of the left proximal femur after the further evaluation and anesthetic clearance of the patient under limb salvage, tumor excision (Figure 5C), and endoprosthesis fixation. The patient was regularly followed up with clinical evaluation and radiographs (Figure 5D), and a significant improvement in the MSTS was recorded.

## Discussion

The proximal femur is a common site for primary bone tumors. Ewing's sarcoma, osteosarcoma, and giant cell tumors are among the common tumors that require adequate medical attention and treatment. Limb salvage surgery is now widely accepted as the most effective treatment option, providing near-normal functionality, high patient satisfaction, and a low complication rate. The limb salvage process involves meticulous evaluation and management, along with adequate limb reconstruction.

Donati et al. [6] conducted a study on 25 patients with proximal femur tumors who were treated with uncemented, bipolar, and modular prostheses. It has been reported that there is persistent pain and limping due to inadequate soft tissue attachment, but the patients have shown 68% satisfactory functional outcomes. Thus, modular endoprostheses have the advantage of adequate soft tissue attachment, which can further enhance their functionality.

In a study conducted by Kabukcuoglu et al. [7], 54 patients with a mean age of 41 years and aggressive tumors of the proximal femur underwent endoprosthetic replacement. Out of these cases, six patients underwent acetabular revision, and seven patients underwent revision of the femoral component. There was a loss of follow-up as 35 patients were still alive at the time of the review, which has its own limitations. The functional outcome score was 83%, indicating successful long-term results during a follow-up period of nine years.

Proximal femoral giant cell tumors (GCT) are benign tumors, yet they are very aggressive in nature. They are located in the metaphyseal-epiphyseal area and are associated with significant bone destruction, causing pathological fractures. Surgery is the mainstay of treatment for resectable GCT, aiming to achieve local tumor control and preserve joint function. In patients with recurrent cases, it is important to consider limb salvage [8]. In our study, we witnessed a case of recurrent GCT in the right proximal femur, which was treated with limb salvage using an endoprosthesis. On regular follow-up, the patient had a satisfactory functional outcome.

In our study, we studied a total of 32 patients with proximal femur tumors. All patients underwent limb salvage surgery and reconstruction with endoprostheses. We identified a few complications, such as infections in four cases (12.5%), dislocation in two cases (6.25%), and aseptic loosening in two cases (6.25%), but there was no documented risk of amputations. The functional outcome was analyzed using the MSTS, and the results showed a good outcome of 72.84%. At the same time, the limitations of our study include a small sample size and a unicenteric assessment.

Many studies have been conducted on endoprosthetic fixation and arthroplasties in proximal femur tumors. Each study has its own advantages and complications, but the primary goal is limb salvage [9-15]. These studies have shown good results. The MSTS [16] is a reliable tool for assessing functional outcomes.

## Conclusions

In conclusion, the use of modular endoprosthesis in proximal femur tumors offers a cost-effective way to enhance the quality of life and functionality of patients by means of a limb salvage option prior to resorting to amputation. Hence, limb salvage should be considered the first modality of treatment to preserve the reasonable functionality of the limb.
